# Correlation between classification in risk categories and clinical aspects and outcomes **^1^**

**DOI:** 10.1590/1518-8345.1284.2842

**Published:** 2016-12-08

**Authors:** Gabriella Novelli Oliveira, Cássia Regina Vancini-Campanharo, Maria Carolina Barbosa Teixeira Lopes, Dulce Aparecida Barbosa, Meiry Fernanda Pinto Okuno, Ruth Ester Assayag Batista

**Affiliations:** 2 Master's student, Escola Paulista de Enfermagem, Universidade Federal de São Paulo, São Paulo, SP, Brazil, Enfermeira, Hospital Universitário, Universidade de São Paulo, São Paulo, SP, Brazil.; 3 PhD, RN, Escola Paulista de Enfermagem, Universidade Federal de São Paulo, São Paulo, SP, Brazil.; 4 MSc, RN, Escola Paulista de Enfermagem, Universidade Federal de São Paulo, São Paulo, SP, Brazil.; 5 PhD, Associated Professor, Escola Paulista de Enfermagem, Universidade Federal de São Paulo, São Paulo, SP, Brazil.; 6 PhD, Adjunct Professor, Escola Paulista de Enfermagem, Universidade Federal de São Paulo, São Paulo, SP, Brazil.

**Keywords:** Triage, Emergency Medical Services, Emergency Nursing, Clinical Evolution, Protocols, User Embracement

## Abstract

**Objective:**

to correlate classification in risk categories with the clinical profiles, outcomes and origins of patients.

**Method:**

analytical cross-sectional study conducted with 697 medical forms of adult patients. The variables included: age, sex, origin, signs and symptoms, exams, personal antecedents, classification in risk categories, medical specialties, and outcome. The Chi-square and likelihood ratio tests were used to associate classifications in risk categories with origin, signs and symptoms, exams, personal antecedents, medical specialty, and outcome.

**Results:**

most patients were women with an average age of 44.5 years. Pain and dyspnea were the symptoms most frequently reported while hypertension and diabetes mellitus were the most common comorbidities. Classifications in the green and yellow categories were the most frequent and hospital discharge the most common outcome. Patients classified in the red category presented the highest percentage of ambulance origin due to surgical reasons. Those classified in the orange and red categories also presented the highest percentage of hospitalization and death.

**Conclusion:**

correlation between clinical aspects and outcomes indicate there is a relationship between the complexity of components in the categories with greater severity, evidenced by the highest percentage of hospitalization and death.

## Introduction

Overcrowding is one of the main problems for Emergency Rooms (ERs) around the world. The causes leading to the increased demand for such services include: difficult access to the health network, increased prevalence of chronic diseases accruing from increased life expectancy, more frequent accidents and urban violence^1^ ^-^ ^2^. In this context, ERs are characterized as one of the main entrance doors into the health system, and cases not characterized as emergencies are the ones that most consume this type of service, due to its convenience and the difficulty individuals face accessing primary health care (PHC) services^3^. One recent South Korean study reports that on days with overcrowding, delay in providing complex care was associated with increased intra-hospital mortality^4^. Therefore, one of the consequences of overcrowding in ERs is the need to identify those who require immediate care among patients, because the time between medical assessment and treatment influences the patients' prognoses. Risk classification (RC), a resource used in ERs and implemented by nurses, emerged as a tool intended to allow recognizing those patients that require care be provided in the shortest interval of time possible^5^. In the 1990s, hospitals in various countries started adopting and improving RC scales to identify patients according to the severity of their conditions^5^. The most well known international scales are: the Emergency Severity Index (ESI), Australasian Triage Scale (ATS), Canadian Triage Acuity Scale (CTAS), and the Manchester Triage System (MTS)^5^.

In 2004, the Brazilian Ministry of Health (MH) devised the QualiSUS Program and the National Humanization Policy, called HumanizaSUS, and initiated the triage process in Brazil denoted *Acolhimento com Avaliação e Classificação de Risco* [Reception with Risk Assessment and Classification]. The idea was that patients classified according to this device would receive care according to the severity of their conditions and no one would be excluded from this process^6^ ^-^ ^7^.

The MH expected some results after the Reception with Risk Assessment was implemented in emergency rooms, including a decrease in the risk of avoidable deaths, extinction of triage performed by non-qualified workers, giving priority to patients according to clinical criteria, shorter waiting times, and the detection of cases that may aggravate if care is postponed^6^ ^-^ ^7^.

Given this context, and after approximately 10 years of RC in Brazil, we realize that research addressing this topic has assessed the relationship between components of classification and outcomes, seeking to meet some of the MH's objectives. In Brazil, the most frequently used and researched risk classification protocol is the Manchester system, which is implemented in most Brazilian states^8^.

Recently, Brazilian researchers concluded that the Manchester system shows that patients classified in categories of greater risk remain in hospitals longer when compared to low-risk categories, indicating that the Manchester is a good clinical predictor for length of hospitalization of patients with more severe conditions^9^. This is similar to another study that shows the Manchester system is a good indicator of risk of death for patients classified as high-risk, as opposed to low-risk patients^10^.

Given this context, this study's guiding question was: What is the relationship existing between the components of clinical aspects, origin, and outcome with the classification of risk categories? Hence, this study's objectives were to correlate CR categories with the clinical profile, outcomes and origin of patients. 

## Method

This analytical cross-sectional study was conducted from April to June 2014 in the Reception with Risk Classification sector of the ER of the São Paulo Hospital, a public university hospital of high complexity located in the south of the city of São Paulo, SP, Brazil. This facility provides care to approximately 4,000 ambulatory patients and another 1,000 patients are cared for in the ER. The population cared for by this service is mainly composed of adult patients covered by the Brazilian Public Health System (SUS). The sector is open 24 hours a day, seven days a week and the assessment is performed by a nurse, who provides a brief nursing consultation in which the patient is asked about signs and symptoms, time of the onset of symptoms, personal history, medications, and allergies. Vital signs are taken and a color code, according to the category of risk, is assigned to the patient, who is then referred to a medical specialty. The RC protocol is institutional and was implemented in 2009 based on the Ministry of Health's guidelines^6^ ^-^ ^7^ and on the Manchester system. This protocol is composed of five categories, identified by colors, while each color refers to a waiting time: red (immediate care); orange (10 minutes), yellow (60 minutes), green (120 minutes), and blue (240 minutes).

The study project was approved by the Institutional Review Board at the Federal University of São Paulo (UNIFESP) (CAAE 05739412910015505). The study population was composed of forms from patients 18 years old or older, classified by the RC sector. The forms were manually completed and were digitally available in the facility's electronic system. Incomplete or illegible forms were excluded. Data were collected after sample size was calculated based on the Chi-square test (Effect Size), with a power of 80% and level of significance at 5%, so that a minimum of 531 electronic forms were established.

Data were accessed online using the institution's system. Data were collected through an instrument developed by the researchers addressing demographic data (age and sex), time the patient arrived at the RC sector, time of medical consultation and discharge, origin (residence, mobile emergency service-SAMU, or ambulatory medical care-AMA), patient's signs and symptoms (classified into respiratory symptoms, pain, bleeding, incapacity to move body parts due to musculoskeletal lesion, trauma, nausea, psychiatric symptoms, dermatological and infectious symptoms, neurological symptoms, malaise and vomiting, abdominal symptoms, gestational or other symptoms), complementary exams (electrocardiogram, image and laboratorial exams) and personal history (high blood pressure, *diabetes mellitus*, heart disease, stroke, smoking, alcohol consumption, cancer, pregnancy, or others), RC category (red, orange, yellow, green or blue), medical specialty (cardiology, surgery, medical clinic, gynecology, neurosurgery, neurology, orthopedics, ENT, and psychiatrics), and the outcome after care delivery (discharge, hospitalization, or death).

All the variables were stored in Microsoft Office Excel^(r)^ spreadsheets, version 2003, for statistical analysis.

A total of 696 out of the 813 forms collected were analyzed: 117 forms were excluded due to being illegible or incomplete. The Statistical Package for the Social Sciences (SPSS), version 19 was used for the analysis.

Categorical variables were descriptively (frequency and percentage) analyzed, while mean, standard deviation, median, minimum and maximum values were calculated for the continuous variables. Chi-square, or when necessary, the likelihood ratio test, was used to compare RC with origin, primary complaint, signs and symptoms, complementary exams, personal antecedents, medical specialty, and outcomes, with a level of significance at 5% (p-value < 0.05).

## Results

The 696 forms showed a predominance of female patients (n = 418; 60.1%), aged 44.5 (±19.2) years old on average, while the origin of most patients was residence (n = 682; 98.0%). The most frequent signs and symptoms were: pain (n = 304; 44.1%), dyspnea (n = 97; 14.1%), incapacity to move a body part due to musculoskeletal lesions (n = 89; 13.0%), and dermatological and infectious problems (n = 79; 11.4%). In regard to personal history, the most prevalent morbidities included systemic blood hypertension (n = 119; 18.1%) and *diabetes mellitus* (n = 51; 7.8%). Most patients were classified in green (n = 422; 61.1%) or yellow categories (n = 151; 21.9%) and were referred to the following specialties: medical clinic (n = 255; 36.8%), orthopedics (n = 116; 16.8%), and surgery (n = 91; 13.2%). The exams most frequently ordered were imaging (n = 216; 40.4%) and laboratory exams (n = 164; 30.7%). The most frequent outcome was discharge (n = 552; 94.5%).

Most patients classified as red originated from SAMU and AMA. The care required by these patients was mostly due to surgical needs when compared to other risk categories. Patients classified as orange or red presented the highest percentage of hospitalization and death (Table 1).

**Table 1:**
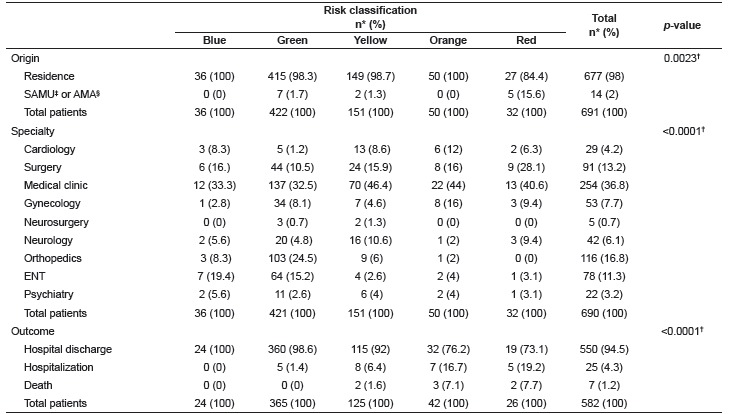
Association between origin, specialty and patient outcomes with risk categories. São Paulo, SP, Brazil, 2015

* Percentage; ^†^ Likelihood ratio test; ^‡^ SAMU: mobile emergency service; ^§^ AMA: Ambulatory medical care

The analysis of signs and symptoms according to the RC showed that patients classified in the red category presented the highest percentage of trauma and lowest percentage of pain; those classified in the orange category presented the highest percentage of respiratory symptoms; and those classified green presented the highest percentage of incapacity in moving a body part due to a musculoskeletal lesion. Patients with gestational symptoms were more frequently classified red or orange (Table 2). The remaining signs and symptoms did not present statistically significant differences in regard to the categories of risk.

**Table 2:**
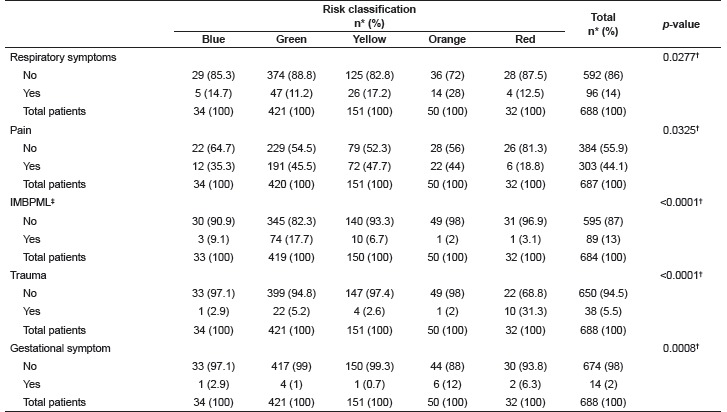
Association of patient signs and symptoms and risk categories. São Paulo, SP, Brazil, 2015

* Percentage; ^†^ Likelihood ratio test; ^‡^ IMBPML: Incapacity in moving a body part due to musculoskeletal lesion

In regard to the exams undertaken by the patients during their stay in the hospital, those classified in the yellow, orange, and red categories required more exams than the others. Electrocardiogram was the most frequent exam among patients classified in the red category, bearing in mind that those classified yellow, orange or red presented the highest percentage of laboratory and image exams (Table 3).

**Table 3:**
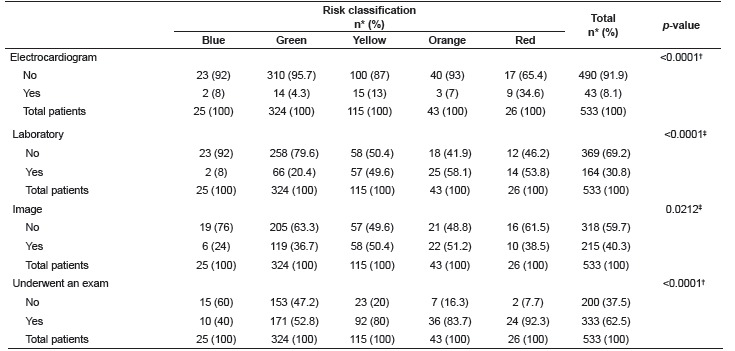
Association of exams conducted during the patients' stay at the hospital and risk categories. São Paulo, SP, Brazil, 2015

* Percentage; ^†^ Likelihood ratio test; ^‡^ Chi-square

When the RC was compared to the patients' personal history, those classified in the yellow, orange, and red categories presented the largest number of antecedents. Patients classified in the blue or green categories presented the lowest percentage of heart disease; patients classified yellow and orange presented the highest percentage of cancer (Table 4). The remaining personal antecedents had no statistically significant association with risk categories.

**Table 4:**
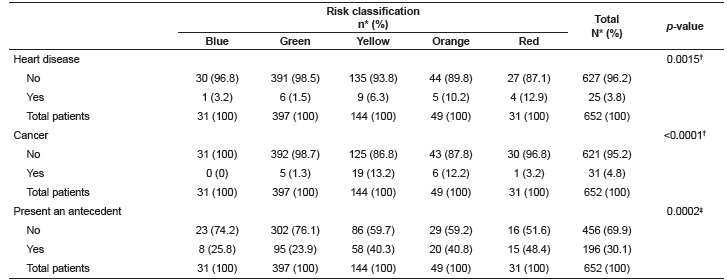
Association of the patients' personal history and risk categories. São Paulo, SP, Brazil, 2015

*Percentage; ^†^ Likelihood ratio test; ^‡^ Chi-square

## Discussion

Overcrowding of ERs in recent years made RC scales a mandatory tool in these facilities^5^. Some of the characteristics of the patients in this study, such as being mostly women (60.1%) with an average age of 44.5 years, are similar to those found in another study addressing forms from the ER of the Odilon Behrens Municipal Hospital in Minas Gerais, Brazil^1^.

In terms of signs and symptoms, pain (44.1%) and dyspnea (14.1%) were the symptoms most frequently presented in the sample. The literature has shown that pain is one of the main complaints presented by individuals seeking care at an ER, with approximately 80% of the patients, even though proper management of pain remains a challenge for emergency services^1^ ^,^ ^11^ ^-^ ^13^.

A Brazilian study, conducted in the ER of a university hospital in the interior of the state of São Paulo, Brazil, and a study conducted in the United States found that systemic arterial hypertension and *diabetes mellitus* presented the highest incident in the populations under study, a result that corroborates this study's findings, as these were the comorbidities most frequently reported, 18.1% and 7.8%, respectively^11^ ^,^ ^14^. These diseases increased in recent years due to population aging, which when associated with sedentariness and obesity, impacted metabolic and cardiovascular diseases and became public health problems^15^. Medical expenditures from complications arising from these diseases are high and patients are increasingly seeking emergency rooms due to clinical decompensation^14^ ^-^ ^15^.

In regard to RC categories, most patients (61.1%) were classified in the green category, followed by yellow (21.9%), showing a tendency of low-risk patients to seek emergency services^1^ ^,^ ^11^; that is, the profile of patients seeking ERs is of low complexity. The reasons are multi-factorial and are linked to a culture of solving problems rapidly, of using technology to perform examinations, easily accessing a service provided 24 hours a day, and geographical location. Due to these factors, hospitals have become the main facility where health care is delivered to the population^3^ ^,^ ^16^.

In this study, the following specialties provided the most care: medical clinic (36.8%), orthopedics (16.8%) and surgery (13.2%). A more intense search for these specialties may be associated with a decompensation of chronic diseases, violence, and traffic accidents^2^ ^,^ ^12^. Another reason is that, up to the present, there is no regulation in Brazil for the medical specialty of urgent and emergency care. Medical teams working in ERs are guided by the urgent and emergency care policies according to the level of complexity of the care delivered. Hence, the nurse implementing RC takes into account the patient's complaint and associates it with signs and symptoms, and refers the patient to a medical specialty accordingly^2^.

The most frequently demanded diagnostic exams were imaging (40.4%) and laboratory exams (30.7%). Exams play an important role in emergency services because they help to establish a medical diagnosis, though the wait for exam results extends the time of the patient in the sector, contributing to the overcrowding in these units. Nonetheless, in this context of high demand, the release of medical reports may be delayed, just as there may be failures in the production process of the imaging sector and such delays and failures may harm patients. Various diagnostic exams, such as laboratory exams, can be performed in PHC units, minimizing potential failures that are observed on overcrowded days^11^ ^,^ ^17^.

Hospital discharge (94.5%) was the most frequent outcome, similar to what other studies describing the profile of patients seeking emergency services in the south and southeast of Brazil, have reported. This outcome is likely to be related to the low complexity of the patients' clinical conditions, indicating that these patients could have been cared for in a PHC service, as well as showing a preference on the part of the population to seek emergency services^9^ ^,^ ^11^.

Those classified in the red category presented the highest percentage of patients originating form SAMU and AMA, and mostly demanded care due to surgical reasons when compared to other risk categories. This may be associated with the hospital's urban locale, which is near major traffic routes, with a high incident of vehicle accidents, as the patients classified in the red category presented the highest percentage of trauma. Although one study conducted in Rio de Janeiro assessing SAMU's regulation reports a predominance of clinical care^18^.

Therefore, we can infer that RC in ERs is a tool guiding the management of SUS because it enables the assessment of patients needs, with patients being classified in the green or blue categories, as they present low complexity conditions, but their conditions were not resolved in a PHC service due to the limited capacity of care in those services^19^.

Patients with more severe conditions, classified in the orange or red categories, presented the highest percentage of hospitalization and death. This finding partly corroborates another study conducted in the ER of *Santa Casa de Caridade de Diamantina* (MG), in which more deaths were verified among patients classified as severe: 42.8% red; 17.0% orange; and 8.9% yellow^9^. The way patients progress is different among risk categories and those classified in the red category are clearly more severe and more prone to the occurrence of death^9^ ^-^ ^10^. The literature has discussed the factors related to the hospitalization and death of patients coming from ERs. The length of time they stay in these places, overcrowding, the presence of chronic diseases, and level of severity, have been observed as potential associated factors^4^ ^-^ ^5^ ^,^ ^10^.

In the analysis of signs and symptoms according to risk category, patients classified in the red category presented the highest percentage of trauma. It may be associated with the Brazilian epidemiological profile, in which external causes are among the primary causes of mortality and morbidity in the last four decades, representing the second most frequent cause of death in Brazil; accidents and homicides are the primary cause of this increase^20^. Additionally, as previously mentioned, the facility's geographical location may have contributed to this result.

Incapacity to move a body part due to musculoskeletal lesion was significantly associated with the green category, when compared to other signs and symptoms. This result may be associated with the greater prevalence of orthopedic problems as one of the most common reasons that patients seek ERs, while lower back pain is one of the most frequently mentioned in the literature^21^. Gestational symptoms were more frequently reported by patients classified as orange or red, which may be associated with the severity of patients cared for in the São Paulo Hospital, considering this is a reference service for high-risk pregnancies^22^.

In regard to the exams patients underwent during their stay at the hospital, those classified as yellow, orange, or red received more exams in comparison to the other categories, which may be explained by the greater severity of their conditions and the need to undergo more exams to establish a diagnosis and treatment, which reflects the facility's constant need to invest in equipment and material^23^. Electrocardiogram was the most frequently performed exam for patients in the red category, indicating this study's patients reported chest pain and, consequently, were considered high-risk patients, as time is essential for a good prognosis in these cases, regardless of the risk classification protocol; a delayed diagnostic in the case of an acute myocardial infarction increases the risk of complications and death^24^.

Analysis of the relationship between the patients' personal history and risk categories shows that those classified in the yellow, orange, or red categories presented a greater percentage of personal antecedents. This may be related to the complications of chronic diseases, which may be severe or fatal and, for this reason, leads patients to seek ERs more frequently^2^ ^,^ ^14^. One study conducted in the United States reports that the frequency with which patients seek emergency services was associated with at least one comorbidity, and these individuals make from four to six visits to an ER in a year^14^. Additionally, a higher percentage of cancer patients was observed in the yellow and orange categories, which may be associated with the side effects of oncological treatments, also leading these patients to more frequently seek emergency service^25^.

RC is a tool that is necessary to organizing the flow of care provided in an ER and enabling more efficient problem-solving capacity and the delivery of humanized care to those in situations of risk^8^. Nevertheless, after RC is applied, and depending on the scale used, for various reasons, patients are not reclassified until a physician assists them - and often, due to the delay between the classification and medical attention, their clinical condition may deteriorate. Correlating RC categories with clinical profile and outcomes is necessary in this context as one of the measures to solve the problem of overcrowding in ERs. 

This study's limitation is the impossibility of comparing results with those from another facility and the fact this protocol has not been validated yet. 

## Conclusion

There is a correlation between the RC categories and the components of clinical aspects, outcomes and origins of patients. The high-risk categories (orange and red) presented the highest percentage of hospitalizations, deaths, exams, comorbidities, and most of these patients were brought in by SAMU. These correlations indicate there is a relationship between the complexity of clinical aspects, outcomes and origins in the cases of greatest severity.

Therefore, managing the flow of patients is not an easy task. Recognizing the relationship between the severity and complexity of cases supports decision-making and proper management of emergency cases. To that end, studies have shown that RC assumes a role beyond the assessment of the severity of one's condition, as a tool that enables researchers to investigate the dynamics of emergency services.
